# Detection and Persistence of Human Semen and DNA on Animal Fur: Implications for Forensic Investigation of Animal Sexual Abuse

**DOI:** 10.3390/ani16203212

**Published:** 2026-10-13

**Authors:** Khilona Natha, Calvin Gerald Mole, Laura Jane Heathfield

**Affiliations:** Division of Forensic Medicine and Toxicology, Department of Pathology, Faculty of Health Sciences, University of Cape Town, Anzio Road, Observatory, Cape Town 7925, South Africa; nthkhi001@myuct.ac.za (K.N.); calvin.mole@uct.ac.za (C.G.M.)

**Keywords:** animal cruelty, sexual offences, body fluid identification, DNA profiling, semen retention, time since intercourse

## Abstract

Animal sexual abuse (ASA) involves the sexual molestation of animals by humans. Forensic DNA analysis of human biological fluids on animal fur may provide investigative leads to a potential suspect. This study was the first to longitudinally assess the recovery of human semen and DNA from three fur models: freshly dissected baboon fur, preserved antelope hides and faux fur. Daily swabbing consistently demonstrated that DNA profiles could be generated from semen over the 14-day study period. Samples in baboon fur typically had greater degradation and yielded lower DNA concentrations compared to other fur models investigated. Although the baboon model best represented a deceased animal, experiments beyond 14 days were limited by decomposition. This study highlights the challenge of selecting a suitable fur model and demonstrates that model choice may influence DNA retention over time. Future research should consider models representing living animals and investigate dynamic variables (e.g., movement, body heat) on semen and DNA retention on animal fur. Overall, this study provides proof of concept and constitutes the first baseline data pertaining to evidence retention in the context of ASA. These findings support the application of forensic molecular techniques in ASA investigations.

## Simple Summary

Animal sexual abuse (ASA) is a serious but poorly understood crime. Investigating ASA can be difficult, owing to limited information about how long evidence of sexual contact remains on an animal. This study aimed to determine whether standard forensic methods used to detect semen and identify human DNA could also be used on animal fur, and to establish how long this evidence could be recovered after contact. Human semen was applied to three types of fur: baboon fur, preserved nyala fur and synthetic fur, and examined over 14 days. Semen was successfully detected on all three types of fur throughout the study period. Human DNA was also recovered from all fur types, and the resulting DNA profiles were of sufficient quality to identify the semen donors. The type of fur affected how much DNA could be recovered, with baboon fur generally producing lower amounts of DNA, with lower quality. The study demonstrates that forensic molecular testing can provide valuable evidence in ASA investigations, even when some time has passed. These findings provide important baseline information for future research and could help veterinarians and forensic investigators collect and interpret evidence, establish timelines and support legal investigations and prosecutions.

## 1. Introduction

Animal sexual abuse (ASA) involves harm inflicted on animals for human sexual pleasure [1]. This can involve fondling of genitalia, vaginal, anal or oral penetration, oral–genital contact, penetration with an object and the injuring or killing of an animal for sexual gratification [1]. Men more commonly partake in the act, with dogs and horses more frequently abused [1,2,3,4,5]. Other sexually abused animals include goats, sheep, chickens and cows [1].

In a study on non-accidental injury in animals in the United Kingdom, an ASA prevalence of 6.3% was reported [4]. The occurrence of ASA in other cohort-based studies is either outdated or reported as a secondary behaviour to a primary concern, such as criminal behaviour or psychological disorders [1,6,7,8,9,10,11,12,13,14,15,16]. Overall, determining the true prevalence of ASA is challenging due to under-reporting.

ASA is illegal in many countries and warrants criminal and forensic investigation [1,15,17,18,19,20]. Currently, investigation and confirmation of ASA relies on confessions, eyewitness reports and veterinary forensic examination of abused animals [1,4]. Guidelines for the medico-legal examination of ASA have been provided by Stern and Smith-Blackmore [1]. Evidence of sexual contact as well as the identification of the assailant are based on body fluid identification (BFI) and DNA analyses, respectively. Although blood and saliva are good sources of DNA that can be used for identification, semen has the additional ability to prove some form of sexual activity occurred.

Additionally, data regarding the persistence and recovery of human semen over time can provide guidelines for estimating time since intercourse (TSI) or at the very least time since semen deposition. Based on the interpretation of the forensic evidence obtained, a timeline of events can be constructed and witness statements corroborated. As a result of large-scale cohort data, guidelines for determining the most probable TSI have been published for use in human sexual offence investigations [21,22]; however, no such data exists in the context of ASA. Although case reports of ASA exist, there is a complete absence of prospective research exploring the extent of human semen persistence and recovery from animals over time. This study therefore aimed to investigate if conventional BFI and DNA analyses could be applied in the context of ASA, and to establish baseline data for semen retention over time. Additionally, this study also aimed to contribute to the development of a suitable animal model for use in ASA research. Demonstrating a proof of concept and developing a workflow would subsequently facilitate the investigation of vital research questions relating to this important, yet severely under-researched, field.

## 2. Methods and Materials

### 2.1. Samples

In order to prospectively study the longitudinal retention of human semen on animal fur, a suitable fur model was needed. Although observing the retention of human semen on a living animal’s fur would have been the most representative of an instance of ASA, legal and ethical considerations motivated for the use of a fur model. As a first attempt at contributing towards bridging this research gap, three fur models were assessed: (i) untreated fur from deceased animals; (ii) fur from hunted animals preserved with salt and (iii) faux fur manufactured for use in clothing, upholstery or carpeting.

**Figure 1:**
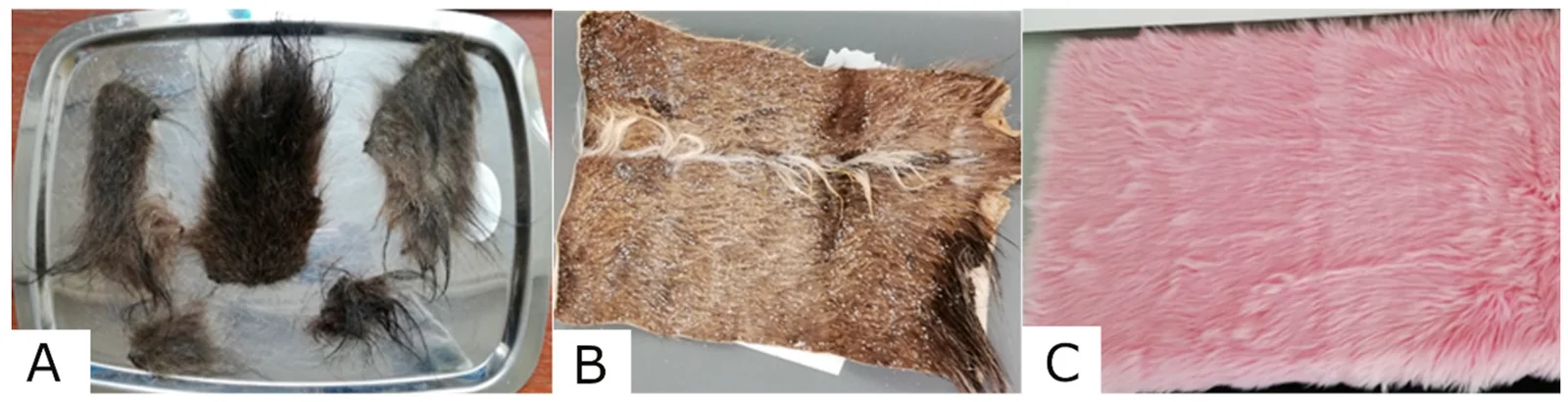
The three fur models used in this study. (**A**) Untreated, real animal fur from a Chacma baboon (*Papio ursinus*); (**B**) treated and semi-processed fur from nyala (*Tragelaphus angasii*); (**C**) faux fur.

Fur was obtained from single individual animals. Untreated fur was obtained from a Chacma baboon head (*Papio ursinus*) (Figure 1A) which was already available in the laboratory. The use of baboon fur is not necessarily representative of all animal fur but was deemed suitable for this study as it is maintained the authenticity of fur from a real animal. The fur was intact and no decomposition of the tissue was present. The fur was dissected from the lateral aspect of the frontal process to the nuchal crest of the baboon head.

Semi-processed fur was of antelope origin, specifically nyala (*Tragelaphus angasii*) (Figure 1B). Excess salt was brushed off prior to experimentation. Lastly, faux fur (Figure 1C) was purchased from a local home decor store and did not require any preparation. Approval was obtained to utilise the baboon and nyala fur (AEC: 019_016). No animals were killed for the purpose of this research.

Additionally, ethical approval for semen collection was obtained (HREC: 190/2019) and two human semen donors were recruited with written informed consent. Exclusion criteria were participants who had a vasectomy, were oligo/azoospermic or had or may have a sexually transmitted disease. Approximately 10 mL of semen from each donor was collected into conical centrifuge tubes, which were then stored at 4 °C.

### 2.2. Preparation and Sampling

The use of unpreserved baboon fur presented a limiting factor on how long experimentation could be conducted for. An initial experiment determined that a 14-day period would be feasible before decomposition of the fur model would make sampling of semen problematic. Semen ejaculates from the same donor were homogenised prior to aliquoting onto each fur model. A total of 28 aliquots, of 100 µL of semen each, were spotted onto each fur model, and allowed to air dry. This allowed for one set of semen stains to be swabbed for BFI purposes and the second set for DNA analyses for the 14-day period. Furs were kept stationary at room temperature in nets to prevent colonisation by insects, and were not exposed to direct sunlight or water.

Prior to each swabbing, an alternative light source (ALS) was used to assist in locating the semen stains. Orange goggles were paired with a Crime-lite™ (Foster and Freeman, Evesham, UK) blue light (430–470 nm) to visualise stains on the nyala and faux fur. Stains on the baboon fur were better visualised using a blue-green light (460–510 nm).

Nylon-flocked swabs (Copan, Brescia, Italy) were moistened with two drops of molecular-grade water and rotated, with the application of a gentle downward force, over the semen stain four times. Swabs for BFI were allowed to air dry for 20 min prior to testing, while the swabs for DNA analyses were immediately processed. The stains for “Day 1” were swabbed after one hour of semen application, and subsequent swabs were taken at 24 h intervals until “Day 14”. Background swabs from areas absent of semen were also taken from each of the furs, and underwent the same analyses as described below.

### 2.3. Body Fluid Identification

Semen was extracted from swabs using 350 µL of 1 X phosphate-buffered saline solution and subsequent incubation at room temperature (21–23 °C) for five minutes. Samples were then vortexed for 15 s followed by centrifugation at 17,900× *g*. Excess liquid was squeezed from the swabs and the swabs were discarded. Semen extracts were centrifuged again as above. The supernatant (200 µL) was separated from the pellet and stored in a clean microcentrifuge tube for presumptive testing (PT), whereas the pellet was retained for confirmatory testing (CT).

PT was achieved by using Brentamine Fast Blue to test for the presence of acid phosphatase (AP). The Brentamine Fast Blue reagent was prepared as previously described [23]. Subsequently, 50 µL of Brentamine Fast Blue solution was added to an aliquot (60 µL) of the supernatant from the extracted swabs. The time it took for a colour change to purple was recorded, with the cut-off time for a positive reaction being ten minutes [24]. This was done in triplicate for each of the samples.

For CT, the pellet was resuspended in the remaining supernatant and 5 µL was spotted onto microscopy slides (Sysmex, Norderstedt, Germany), heat-fixed and stained with haematoxylin and eosin. One slide per swab extract and a positive control from each donor were prepared. Slides were viewed using a Leica DM500 microscope (Leica, Wetzlar, Germany) with a ICC50 HD camera attachment (Leica, Wetzlar, Germany) and 400× magnification. Spermatozoa abundance was scored according to Allard [22] and Nolan et al. [25].

### 2.4. DNA Analyses

DNA was extracted from semen swabs using the QIAamp^®^ DNA Investigator Kit (Qiagen, Hilden, Germany) following the manufacturer’s protocol (page 13–16). However, 0.1% (6.5 mM) dithiothreitol (DTT) was added to the first lysis step. DNA was eluted into 100 µL of ATE buffer. DNA was also extracted from 100 µL of neat semen from each of the donors.

To quantify DNA, qPCR was conducted using the Investigator Quantiplex^®^ Pro Kit (Qiagen, Hilden, Germany) following the manufacturers’ protocol with no deviations (page 13–23). The Applied Biosystems 7500 real-time PCR system (Foster City, CA, USA) was used, and data was captured on HID Real-Time PCR Analysis software v1.2 (Applied Biosystems, Foster City, CA, USA).

For STR DNA profiling, DNA was diluted to 0.5 ng/µL using molecular-grade water based on the concentration of the 91 bp marker (small autosomal marker) quantified in qPCR. The PowerPlex^®^ ESI 16 kit (Promega, Madison, WI, USA) was used for multiplex PCR following the manufacturer’s protocol (pages 6–9) and performed in a T100 Thermal Cycler (Bio-Rad, Hercules, CA, USA). Capillary electrophoresis was achieved using the Applied Biosystems 3130*xl* Genetic Analyzer (Applied Biosystems, Foster City, CA, USA) following the manufacturer’s protocol (pages 27–29). Modifications included using a 50 cm array and a performance-optimised polymer 7 (POP7) and conducting electrophoresis at 15 kV. GeneMapper^®^ version 4.1 software (Applied Biosystems, Foster City, CA, USA) was used to analyse the electropherograms. Positive and negative controls were included throughout experiments and reference profiles were generated from the extracted neat semen samples of the donors.

### 2.5. Data and Statistical Analysis

The dataset analyses in this study comprised observations from two semen donors, with deposition on three fur models (Baboon, Nyala and Faux), measured across 14 sampling days. Baboon and nyala fur models were obtained from single individual animals.

BFI success (i.e., PT and CT outcomes) and DNA metrics (i.e., DNA concentration, degradation and STR profiling success) were assessed against time and animal fur model. For PT, the average time taken for a positive reaction to occur was calculated for each of the replicates. qPCR results were used to assess the quality and quantity of DNA extracted from the semen samples in terms of the degradation index (DI) and DNA concentration. DI was calculated by dividing the concentration of the small autosomal marker (91 bp) by the concentration of the larger autosomal marker (353 bp).

Changes in DNA concentration and degradation index (DI) over the 14-day period were assessed descriptively, with observations presented separately for each semen donor and fur model. Spearman’s rank correlation coefficients were used as an exploratory measure of the association between sampling day and each DNA metric within individual donor–fur model combinations. Spearman rank correlation was conducted using R Studio Version 3.4 and a level of significance of α = 0.05.

Because each fur model was represented by a single individual animal, repeated samples obtained from that substrate were not considered independent biological replicates of the fur model. Consequently, inferential statistical comparisons between the three fur models were not performed. DNA concentration and DI were instead compared descriptively across the fur substrates, with donor-specific observations presented to demonstrate the variability between donors and over time.

The ability to obtain good quality, informative STR DNA profiles from semen stains on animal fur was assessed. A good quality DNA profile was one which had easily discernible peaks at all loci which met the internal analytical (32 RFU) and stochastic thresholds (106 RFU). A DNA profile was regarded forensically informative if it held sufficient discriminatory power for identification purposes, and internally, this was defined as twelve fully typed STRs. Allele calling accuracy was determined by comparing the electropherograms of samples obtained from the furs to the reference DNA profile from the donors. The electropherograms were also compared to profiles generated from the background swabs to evaluate possible interferences arising from animal DNA or other contaminants from the furs.

## 3. Results

Data obtained from the BFI and DNA analyses for all samples and controls can be found in Supplementary Table S1.

### 3.1. Presumptive and Confirmatory Testing

All semen samples from all fur models for the entire two-week period produced a positive reaction. The majority of the samples reacted immediately with a maximum reaction time of 32.67 s (Day 5, baboon fur, donor 1). The time taken for a positive reaction to occur was not associated with the age of the sample, nor fur model. None of the background fur swabs yielded a false positive result.

**Figure 2:**
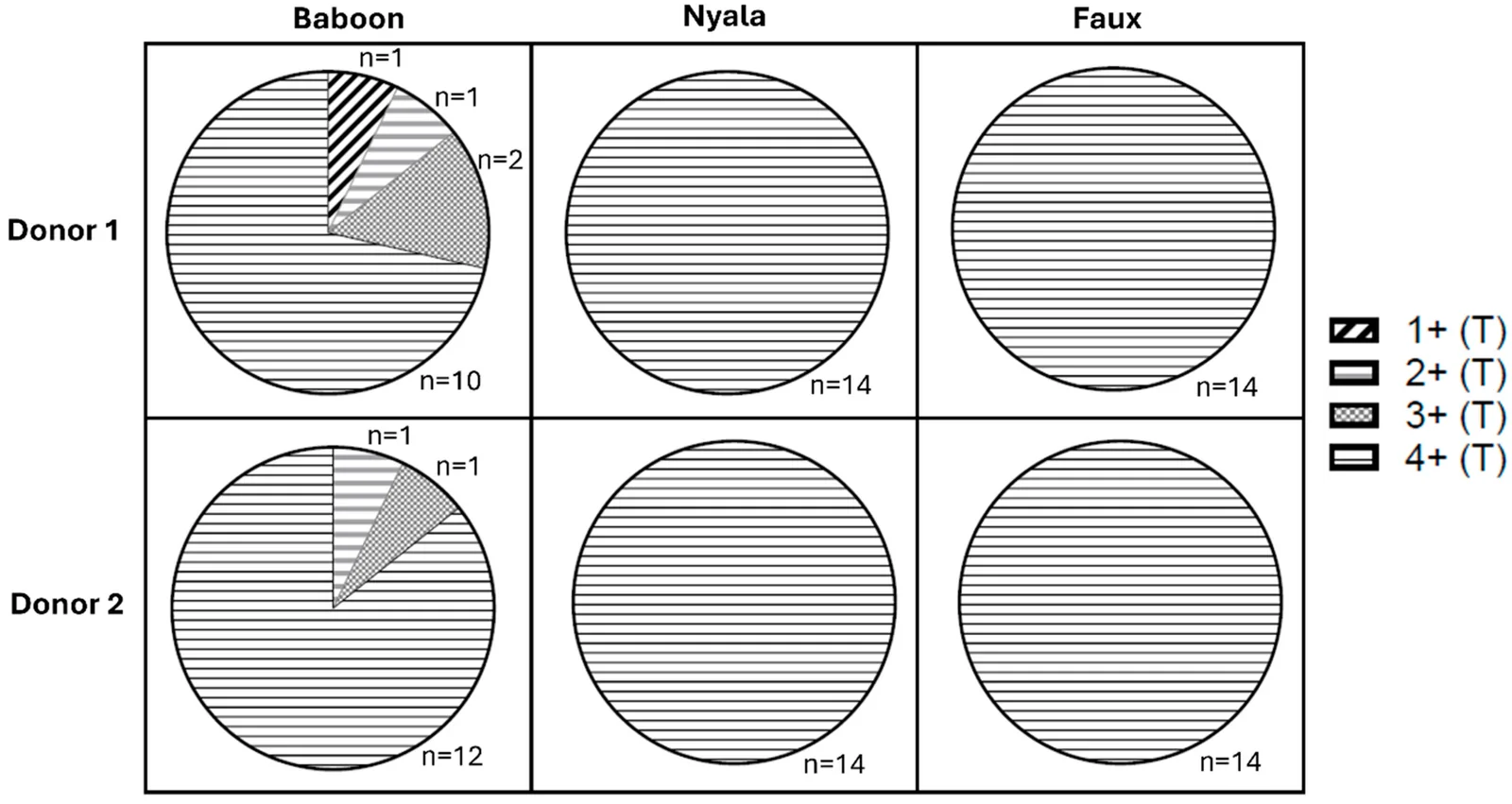
The frequency of spermatozoa scores for each of the donor–model combinations for the entire two-week period. Horizontal black lines indicate a score of 4+ (T), dots represent a score of 3+ (T), horizontal grey lines 2+ (T) and diagonal lines 1+ (T).

Spermatozoa were also observed for all semen samples from all fur models across the entire 14-day period (Supplementary Figure S1). The distribution of spermatozoa scores can be seen in Figure 2. A score of 4+ (T) was obtained for all samples taken from the nyala and faux furs. Samples from the baboon fur showed more variation with lower scores obtained at random time points. Three out of the six samples with scores lower than 4+ (T) correlated with non-immediate AP reaction times (Supplementary Table S1).

### 3.2. DNA Analyses

**Figure 3:**
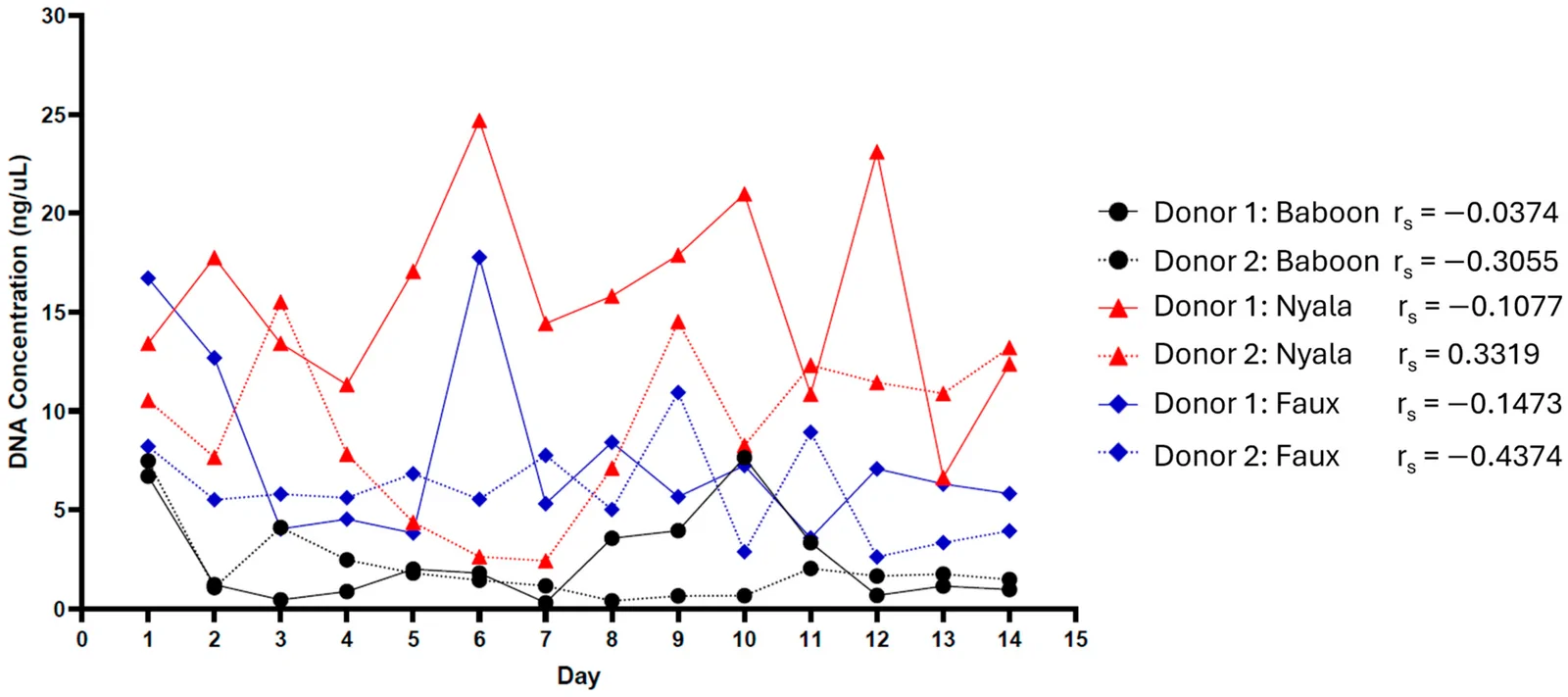
qPCR DNA concentrations (ng/µL) of the small autosomal marker (91 bp) obtained from nyala (triangles), faux (diamonds) and baboon (dots) furs from donor 1 (sold lines) and donor 2 (dashed lines) semen stains that were swabbed over 14 days. Rho factors (r_s_) from the Spearman’s rank correlation coefficient test are indicated.

Figure 3 illustrates the distribution of DNA concentrations per donor–model combination for the two-week period. DNA concentrations from donor 1 tended to be lower than those obtained from donor 2 semen samples. Generally, higher DNA concentrations were obtained from samples swabbed from nyala fur, followed by faux fur and lastly baboon fur within each semen donor. However, a large decrease in DNA concentration was observed in donor 2 semen samples swabbed from nyala fur between days 5 and 7, followed by a subsequent increase, which was not observed in the other fur models. Within each donor–model combination there were no significant correlations between DNA concentration and time.

**Figure 4:**
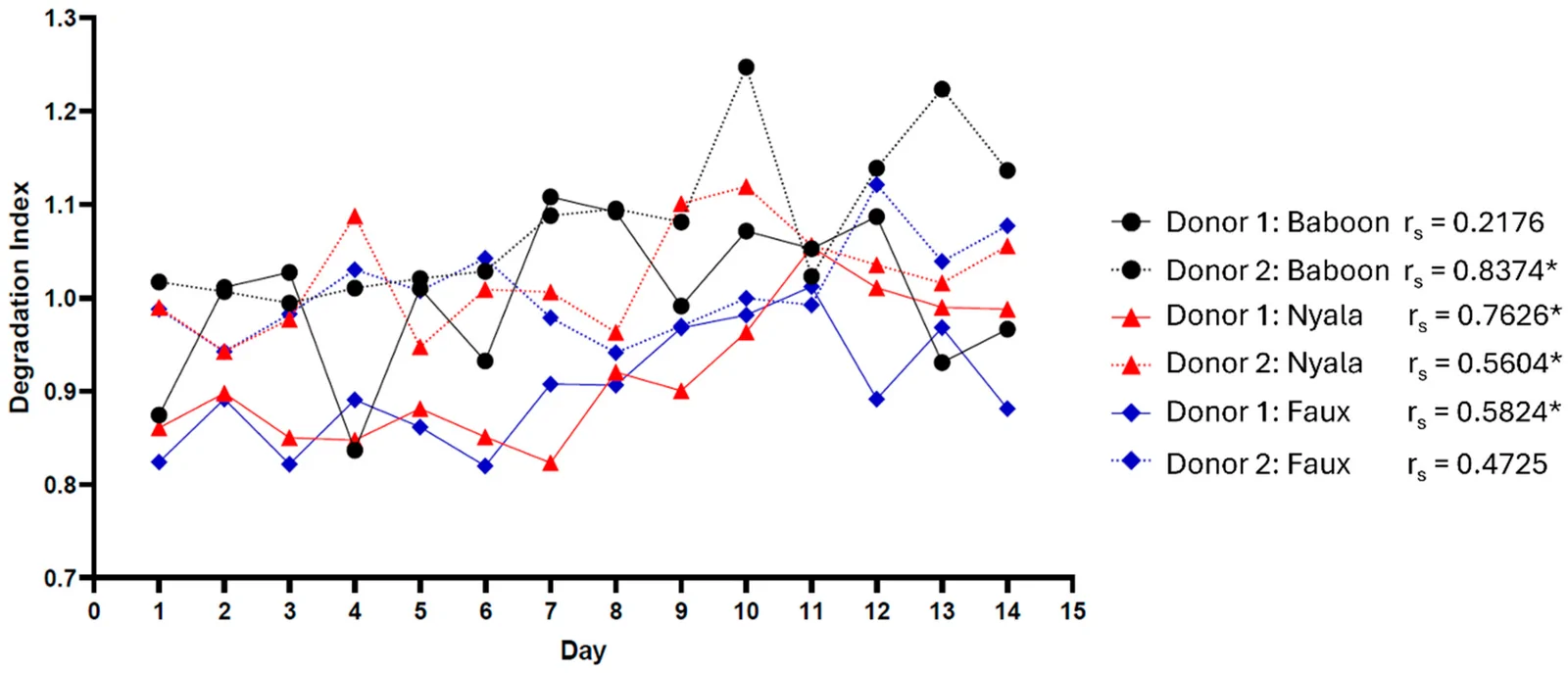
Calculated DI for nyala (triangles), faux (diamonds) and baboon (dots) furs from donor 1 (solid lines) and donor 2 (dashed lines) semen stains that were swabbed over 14 days. Rho factors (r_s_) from the Spearman’s rank correlation coefficient test are indicated and the asterisk (*) denotes a statistically significant relationship between day and DI.

Figure 4 illustrates the DI per donor–model combination over the two-week period. In all donor–model combinations an increase in DI with time was observed with significant positive Spearman’s Rho in all cases except donor1:baboon and donor2:faux. Degradation indices in baboon fur tended to be higher than in other models up until day 11, regardless of semen donor. Degradation indices within nyala and faux fur tended to be similar within samples from the same donors.

The majority of the generated DNA profiles gave a 100% allele call rate. Four samples showed dropout of one to three alleles but were still considered forensically informative. All DNA profiles corresponded to the respective donor’s reference DNA profile. These results indicated sufficient, good quality DNA to produce informative DNA profiles was obtained from the three types of fur for the full two-week period.

The background swabs of the various furs yielded DNA of low concentrations, the maximum being 0.114 ng/µL from baboon fur (Supplementary Table S1). When DNA was detected in background swabs, it was mostly from the small autosomal marker only. These did not translate into peaks on the electropherograms for the nyala and faux furs. However, background swabs from the baboon fur produced several peaks that did not match the donor’s reference profiles, nor any elimination DNA profile from researchers in the laboratory (Supplementary Figure S2B). These comprised two off-ladder peaks at 237 bp and 241 bp at marker D2S1338, allele 7 at 305 bp at marker D22S1045 and allele 15 at 124 bp at marker D16S5539. One to four of these additional peaks were observed in some of the profiles generated from semen samples obtained from the baboon fur (Supplementary Figure S2A,C), but they were substantially lower in relative fluorescent units. These additional peaks were attributed to amplification from the baboon fur itself and could be eliminated based on the profiles obtained from the background swabs of the fur.

## 4. Discussion

This study has demonstrated, for the first time, that conventional forensic biology methods can be successfully applied to analysing the longitudinal retention of human semen evidence on animal fur. BFI was possible and DNA concentrations and integrity were sufficient to obtain forensically informative profiles even after two weeks. DNA degradation increased over time in all models for each semen donor, although the correlation was not significant for donor 1 on baboon fur and donor 2 on faux fur. Fortunately, this did not prevent forensically informative DNA profiles from being obtained even after 14 days. Spermatozoa appear to be resilient and remain morphologically intact over extended periods of time and the presence of protamines instead of histones in the chromatin structure makes spermatozoa DNA more resistant to isolation than in somatic cells [26,27,28,29]. Endogenous nuclease activity can still occur in morphologically intact spermatozoa, but this activity may be suppressed through drying of the semen stains [30,31].

While favourable results were observed in all fur models used, the DNA yields from the baboon fur were typically lower than that from the nyala fur and faux fur models regardless of semen donor (Figure 3). This can be explained, at least in part, by the variation in morphology between the models. For example, it was observed that the semen was readily absorbed into the baboon fur after aliquoting, while it appeared to remain at surface level in the other two models. Absorption into the deeper layers of the fur would likely occur in authentic case examples, and may have made semen recovery more challenging. This mechanism would also explain the longer AP reaction times and lower spermatozoa scores obtained for the baboon fur. The faux fur was thicker than the nyala hide and some absorption may have occurred but not to the extent of baboon fur. Indeed, fur varies across the body of a single animal and also displays both intra- and interspecies differences in terms of texture (smooth or coarse), thickness, number of layers, length and style (e.g., straight or curly) [32]. Domestic species, which are more often the victims of ASA, show variations in hair morphology, especially at the microscopic level [33,34], and this in itself can affect body fluid retention in a forensic context. Mammalian fur strands comprise three morphological regions—the medulla, cortex and cuticle, with the cuticle being composed of a layer of keratinised scales [35]. There are three types of scales: coronal (crown-like) scales, spinous (petal-like) scales and imbricate (flattened) scales [35]. Primates, including humans, have imbricate scales, whereas other animals may have any of the three types [35]. These differences in the morphological characteristics of cuticle scales can possibly affect the retention of spermatozoa. Thus, semen retention parameters should be extrapolated with caution as that observed on the baboon and nyala furs may very well differ in other animals. Although beyond the scope of the study, analysing and investigating these microscopic differences between furs, especially those of probable ASA victims, and the influence thereof on semen retention would be worthwhile.

While the use of preserved animal hides and synthetic materials may be convenient, it may not represent a biologically realistic scenario, and therefore would introduce a bias in molecular analyses. With the nyala furs already dehydrated and preserved in salt prior to the application of semen, cellular and tissue degradation is inhibited and DNA integrity maintained [36,37,38]. The faux fur was composed of 100% acrylic (synthetic) fibres and the absence of inherent biological factors would prevent DNA degradation from occurring in a natural manner.

Given the nature of the baboon fur being dissected and unpreserved, it underwent a decomposition process. This natural process encompasses endogenic, microbial and spontaneous chemical enzymatic reactions, which are also known to contribute to loss of DNA integrity [31,39,40,41,42]. These reasons can explain why DNA degradation was significantly higher on the baboon fur when compared to the other models, and why no significant difference in DNA degradation was observed between the nyala and faux fur (Figure 4). From these results, it appears that intrinsic properties of authentic fur, namely morphology, biotic and abiotic factors, play a role in semen retention and recovery. Despite the baboon model showing the most variation in the BFI results and the lowest DNA yields, it best represented the authenticity of animal fur. Even though the fur characteristics may be different amongst animals, the semen retention data obtained from the baboon fur model may likely represent what can be expected from fatal ASA cases.

Given these results, if a veterinarian is presented with a fatal ASA case, every effort should be made to collect semen evidence even if two weeks has passed since the incident. Compared to human sexual assault investigations, sampling from the furs should make use of an ALS to aid in locating the semen stains. Secondly, background fur swabs of the abused animal, as well as reference samples from the owner and veterinarian, need to be obtained. The swabs are important to eliminate artefacts from the animal’s DNA, as well as to eliminate people who legitimately handled the animal. In this study, additional peaks were observed in the DNA profiles from baboon fur, and were not a result of instrumental artefacts or contamination from researchers in the laboratory. They did not resemble a typical mixed DNA profile, but were rather attributed to amplification of baboon DNA.

This result was not unexpected due to high DNA sequence similarity amongst higher primates [43]. Cross-reactivity for gorillas and spider monkeys has previously been demonstrated for the Promega PowerPlex^®^ ESI 16 STR profiling kit, but no validation data is available for the *Papio ursinus* species [44]. Although the domestic animal species often encountered in ASA [1,2,3] do not cause any major interferences in the DNA analytical techniques used in this workflow [44,45], obtaining background fur swabs is still encouraged.

The results of this study illustrate the potential of using molecular forensics in ASA investigations. Given the baseline data produced here, additional variables to assess the effect thereof on semen persistence over time can now be introduced into the model to represent a more realistic ASA scenario. These include, introducing movement, temperature and humidity changes, as well as brushing or washing the fur to simulate grooming, licking and bathing. Using more semen donors to expand the sample size could validate the relationships observed between time and the various tested variables of this study. Furthermore, having large-scale data may aid in providing guidelines for estimating TSI, much like that seen for human sexual assault investigations [21,22].

This study established a suitable methodology for molecular forensic investigations of ASA. Using animal fur as a substrate, other validation studies can be conducted. These include method specificity to detect human semen in mixtures of different body fluids—either with other human body fluids or those of animal origin. Alternative sampling methods for different fur types can also be investigated. In ASA there may be the problem of multiple offenders and Y-STR profiling will be more useful in differentiating the different donors’ DNA profiles [46]. DNA arising from non-perpetrators can also easily be encountered in ASA and evaluating the use of techniques such as differential lysis and extraction, laser capture microdissection, and SpermElution© for DNA extraction specifically from spermatozoa may be worthwhile [47,48,49,50,51]. Method sensitivity can also be investigated by adjusting the volume of semen and spermatozoa abundance applied to the fur to simulate donor-dependent factors such as ejaculate volume and oligo/azoospermic males.

It must be noted that the detection of semen on fur in ASA cases does not in and of itself indicate sexual abuse or penetration but only indicates semen deposition on the fur which may arise from various sources. Based on the limited sample size and sampling units used in this study, inferences should not be made that the patterns observed in this study will be true in all animal models across all potential semen donors. Nevertheless, the data presented here provides proof of concept for establishing time since deposition and semen donor identity.

## 5. Conclusions

The findings from this study, although limited by semen deposition onto fur from single animals and analysis of data from only two semen donors, provides a valuable proof of concept for the analysis of semen on animal fur in suspected ASA cases. Although there appears to be degradation of DNA over time, the DNA concentration was not dramatically altered across the 14 days. AP was easily detected and abundant spermatozoa were observed for 14 days. Good quality, forensically informative DNA profiles that easily matched the semen donors were also obtained from all fur models. This suggests value in the collection and examination of biological fluids present on animals in suspected ASA cases even if some time has passed since the suspected act. This research has successfully provided baseline measurements, and further exploration into semen persistence on animal fur would aid better inferential conclusions.

## Data Availability

The original contributions presented in this study are included in the article/Supplementary Materials. Further inquiries can be directed to the corresponding author.

## Supplementary Materials

The following supporting information can be downloaded at https://www.mdpi.com/article/10.3390/ani16203212/s1: Figure S1: Examples of microscopic analyses on extracted background swabs (A–C) and swabbed semen stains (D–F, taken on Day 14) observed on the different fur types. Figure S2: Example of the additional peaks in the electropherograms (red circles and red squares) observed from semen stains on the baboon fur (A,C) that corresponded to those observed in background swabs from the baboon fur (B). Figure S3: Flow diagram of experimental procedures. Table S1: Results obtained from all experimental analyses conducted on samples, including background samples, obtained over the 14-day period from both semen donors for all fur models.
