# Supplementary material for: Detection and Persistence of Human Semen and DNA on Animal Fur: Implications for Forensic Investigation of Animal Sexual Abuse
Source: Animals (Basel). 2026 Oct 13;16(20):3212. doi: 10.3390/ani16203212 (PMC13700667; doi:10.3390/ani16203212)
Supplement: Supplementary file 1 [file animals-16-03212-s001.zip › Supplementary data.pdf]

Supplementary data: Data from experimental analyses

Table S1: Results obtained from all experimental analyses conducted on samples, including background samples, obtained over the 14-day period from both semen donors for all fur models. Where applicable, data presented here has been rounded off to four decimal places, however statistical analysis was conducted on raw data. (PT = presumptive testing, CT = confirmatory testing, bp = base pairs, DI = degradation index, * = average time from three replicates, ✓ = immediate colour change to purple, ✗ = no colour change up to ten minutes, s = seconds, (T) = tails, N/A = not applicable).

					DNA concentration (ng/µL)			
**Day**	**Model**	**Participant**	**PT***	**CT**	**91 bp marker**	**353 bp marker**	**DI**	**Profiling success (% of alleles called)**
	Positive control	1	✓	4+ (T)	26.8661	30.7012	0.8751	100
		2	✓	4+ (T)	24.5621	24.6654	0.9958	100
	Baboon	Blank 1	✗	Dirt/Cell material	0.0094	0.0012	7.8245	Some peaks called, few off-ladder peaks
		Blank 2	✗	Dirt/Cell material	0.0091	0.0	N/A	Some peaks called
		Blank 3	✗	Dirt/Cell material	0.1138	0.0	N/A	Some peaks called, few off-ladder peaks
		Blank 4	✗	Dirt/Cell material	0.0078	0.0	N/A	Some peaks called, few off-ladder peaks
		Blank 5	✗	Dirt/Cell material	0.0078	0.0	N/A	Some peaks called, few off-ladder peaks
		Blank 6	✗	Dirt/Cell material	0.0125	0.0	N/A	Some peaks called, few off-ladder peaks
		Blank 7	✗	Dirt/Cell material	0.0163	0.0	N/A	Some peaks called, few off-ladder peaks
	Nyala	Blank 1	✗	Salt	0.0033	0.0029	1.1426	0
		Blank 2	✗	Salt	0.0004	0.0	N/A	0
	Faux	Blank	✗	Dirt	0.0008	0.0005	1.7287	0

Table S1 cont.

					DNA concentration (ng/µL)			
Day	Model	Participant	PT*	CT	91 bp marker	353 bp marker	DI	Profiling success (% of alleles called)
1	Baboon	1	✓	4+ (T)	6.7105	7.6746	0.8744	100
		2	✓	4+ (T)	7.4703	7.3452	1.0170	100
	Nyala	1	✓	4+ (T)	13.4195	15.5937	0.8606	100
		2	✓	4+ (T)	10.5426	10.6502	0.9899	100
	Faux	1	✓	4+ (T)	16.7190	20.2841	0.8242	100
		2	✓	4+ (T)	8.2130	8.3128	0.9880	100
2	Baboon	1	✓	4+ (T)	1.2174	1.2039	1.0112	100
		2	✓	3+ (T)	1.0670	1.0599	1.0067	100
	Nyala	1	✓	4+ (T)	17.7550	19.7749	0.8979	100
		2	✓	4+ (T)	7.6667	8.1344	0.9425	100
	Faux	1	✓	4+ (T)	12.6843	14.2267	0.8916	100
		2	✓	4+ (T)	5.5165	5.8534	0.9424	100
3	Baboon	1	12.33 s	2+ (T)	0.4475	0.4355	1.0274	100
		2	✓	4+ (T)	4.1115	4.1343	0.9945	100
	Nyala	1	✓	4+ (T)	13.4221	15.7895	0.8501	100
		2	✓	4+ (T)	15.5142	15.8774	0.9771	100
	Faux	1	✓	4+ (T)	4.0355	4.9097	0.8219	96.88
		2	✓	4+ (T)	5.8002	5.9030	0.9826	100

Table S1 cont.

					DNA concentration (ng/µL)			
Day	Model	Participant	PT*	CT	91 bp marker	353 bp marker	DI	Profiling success (% of alleles called)
4	Baboon	1	✓	4+ (T)	0.8704	1.0401	0.8368	93.75
		2	✓	4+ (T)	2.4723	2.4470	1.0104	100
	Nyala	1	✓	4+ (T)	11.3358	13.3741	0.8476	100
		2	✓	4+ (T)	7.8210	7.1910	1.0876	100
	Faux	1	✓	4+ (T)	4.5317	5.0884	0.8906	93.75
		2	✓	4+ (T)	5.6168	5.4521	1.0302	100
5	Baboon	1	32.67 s	1+ (T)	2.0008	1.9806	1.0102	100
		2	✓	4+ (T)	1.8095	1.7727	1.0208	100
	Nyala	1	✓	4+ (T)	17.0619	19.3621	0.8812	100
		2	✓	4+ (T)	4.3791	4.6210	0.9476	100
	Faux	1	✓	4+ (T)	3.8278	4.4424	0.8617	100
		2	✓	4+ (T)	6.8309	6.7796	1.0076	100
6	Baboon	1	✓	3+ (T)	1.8090	1.9402	0.9324	90.63
		2	✓	4+ (T)	1.4441	1.4043	1.0284	100
	Nyala	1	✓	4+ (T)	24.6967	29.0240	0.8509	100
		2	✓	4+ (T)	2.6337	2.6104	1.0089	100
	Faux	1	✓	4+ (T)	17.7694	21.6746	0.8198	100
		2	✓	4+ (T)	5.5346	5.3089	1.0425	100

Table S1 cont.

					DNA concentration (ng/µL)			
Day	Model	Participant	PT*	CT	91 bp marker	353 bp marker	DI	Profiling success (% of alleles called)
7	Baboon	1	✓	4+ (T)	0.2996	0.2704	1.1082	100
		2	✓	4+ (T)	1.1557	1.0621	1.0881	100
	Nyala	1	✓	4+ (T)	14.4237	17.5192	0.8233	100
		2	✓	4+ (T)	2.4281	2.4131	1.0062	100
	Faux	1	✓	4+ (T)	5.3158	5.8562	0.9077	100
		2	✓	4+ (T)	7.7610	7.9306	0.9786	100
8	Baboon	1	10.67 s	3+ (T)	3.5637	3.2642	1.0918	100
		2	✓	4+ (T)	0.4017	0.3668	1.0953	100
	Nyala	1	✓	4+ (T)	15.8181	17.1841	0.9205	100
		2	5.33 s	4+ (T)	7.1194	7.3936	0.9629	100
	Faux	1	✓	4+ (T)	8.4206	9.2886	0.9065	100
		2	✓	4+ (T)	5.0128	5.3250	0.9414	100
9	Baboon	1	✓	4+ (T)	3.9569	3.9918	0.9913	100
		2	✓	4+ (T)	0.6531	0.6041	1.0812	100
	Nyala	1	✓	4+ (T)	17.8829	19.8590	0.9005	100
		2	7.33 s	4+ (T)	14.5225	13.1917	1.1009	100
	Faux	1	✓	4+ (T)	5.6594	5.8469	0.9679	100
		2	✓	4+ (T)	10.9352	11.2755	0.9698	100

Table S1 cont.

					DNA concentration (ng/µL)			
Day	Model	Participant	PT*	CT	93 bp marker	353 bp marker	DI	Profiling success (% of alleles called)
10	Baboon	1	5.33 s	4+ (T)	7.6441	7.1335	1.0716	100
		2	✓	2+ (T)	0.6696	0.5368	1.2474	100
	Nyala	1	✓	4+ (T)	20.9788	21.7813	0.9632	100
		2	9 s	4+ (T)	8.2612	7.3800	1.1194	100
	Faux	1	✓	4+ (T)	7.2426	7.3794	0.9815	100
		2	✓	4+ (T)	2.8829	2.8841	0.9996	100
11	Baboon	1	✓	4+ (T)	3.3469	3.1790	1.0528	100
		2	✓	4+ (T)	2.0354	1.9898	1.0229	100
	Nyala	1	✓	4+ (T)	10.8534	10.2948	1.0543	100
		2	✓	4+ (T)	12.3176	11.6630	1.0561	100
	Faux	1	✓	4+ (T)	3.5801	3.5376	1.0120	100
		2	✓	4+ (T)	8.9271	8.9947	0.9925	100
12	Baboon	1	✓	4+ (T)	0.6725	0.6186	1.0871	100
		2	✓	4+ (T)	1.6539	1.4521	1.1390	100
	Nyala	1	✓	4+ (T)	23.1135	22.8680	1.0107	100
		2	✓	4+ (T)	11.4514	11.0609	1.0353	100
	Faux	1	✓	4+ (T)	7.0829	7.9428	0.8917	100
		2	✓	4+ (T)	2.6186	2.3352	1.1214	100

Table S1 cont.

					DNA concentration (ng/µL)			
Day	Model	Participant	PT*	CT	93 bp marker	353 bp marker	DI	Profiling success (% of alleles called)
13	Baboon	1	5.33 s	4+ (T)	1.1471	1.2324	0.9308	100
		2	✓	4+ (T)	1.7519	1.4317	1.2236	100
	Nyala	1	✓	4+ (T)	6.6389	6.7063	0.9899	100
		2	✓	4+ (T)	10.8896	10.7203	1.0158	100
	Faux	1	✓	4+ (T)	6.3135	6.5213	0.9681	100
		2	✓	4+ (T)	3.3477	3.2222	1.0389	100
14	Baboon	1	✓	4+ (T)	0.9678	1.0015	0.9664	100
		2	✓	4+ (T)	1.4801	1.3024	1.1364	100
	Nyala	1	✓	4+ (T)	12.3760	12.5261	0.9880	100
		2	✓	4+ (T)	13.2071	12.5126	1.0555	100
	Faux	1	✓	4+ (T)	5.8194	6.6025	0.8814	100
		2	✓	4+ (T)	3.9380	3.6553	1.0773	100

Supplementary figures: Additional figures from analysis

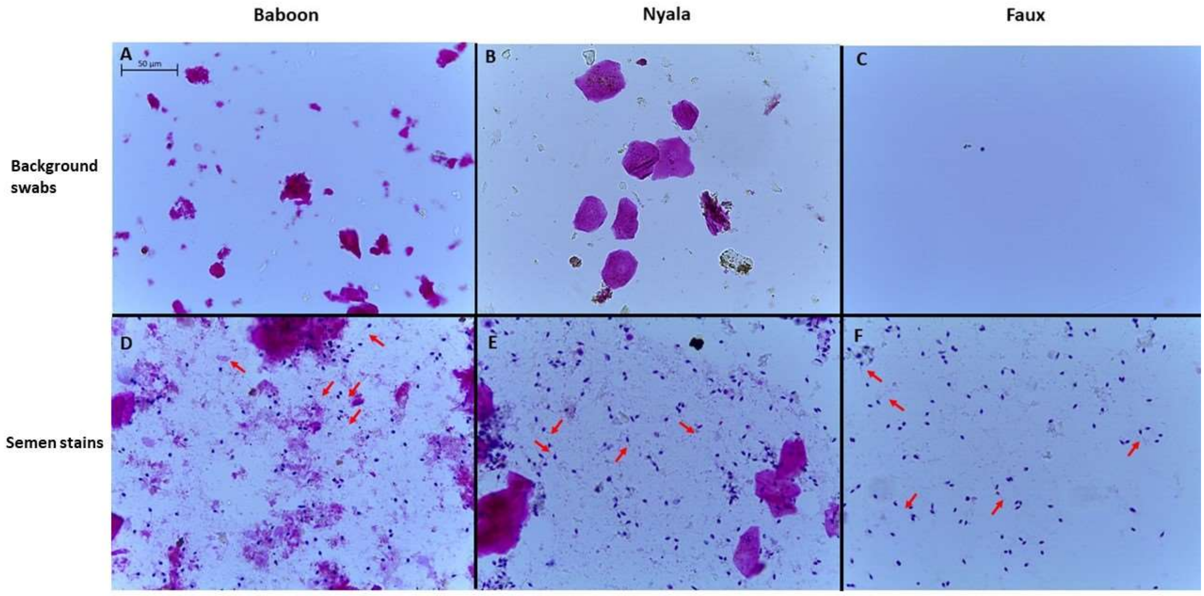

Figure S1: Examples of microscopic analyses on extracted background swabs (A-C) and swabbed semen stains (D-F, taken on Day 14) observed on the different fur types. Red arrows indicate some of the spermatozoa tails. Slides were viewed using a Leica DM500 microscope and 400X magnification. Images were captured with a Leica ICC50 HD camera attachment. Scale bar (A) is equal to 50 µm and is applicable to all images.

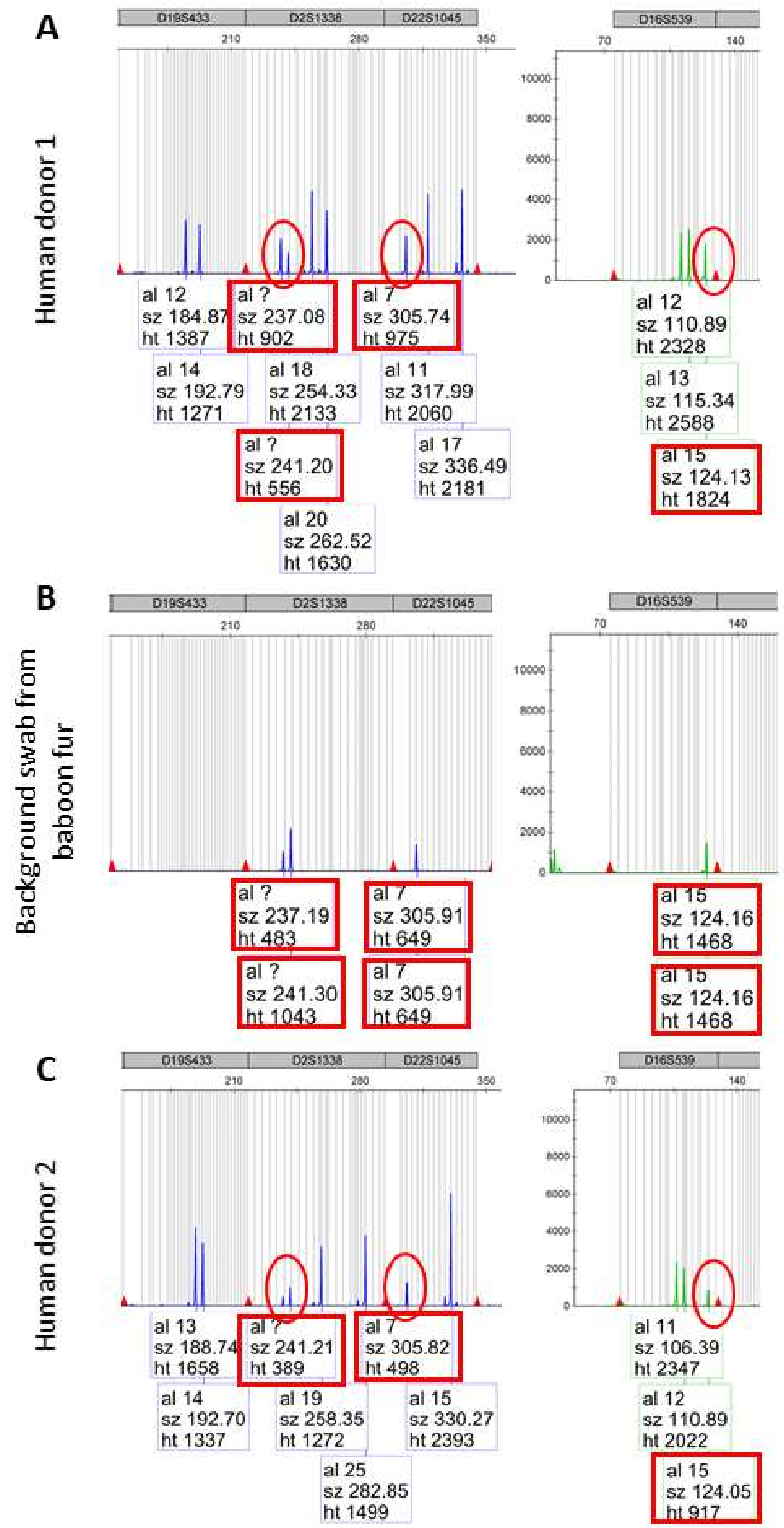

Figure S2: Example of the additional peaks in the electropherograms (red circles and red squares) observed from semen stains on the baboon fur (A and C) that corresponded to those observed in background swabs from the baboon fur (B). Marker size is represented on the x-axis and relative fluorescent units on the y-axis, and each marker is labelled on the top.

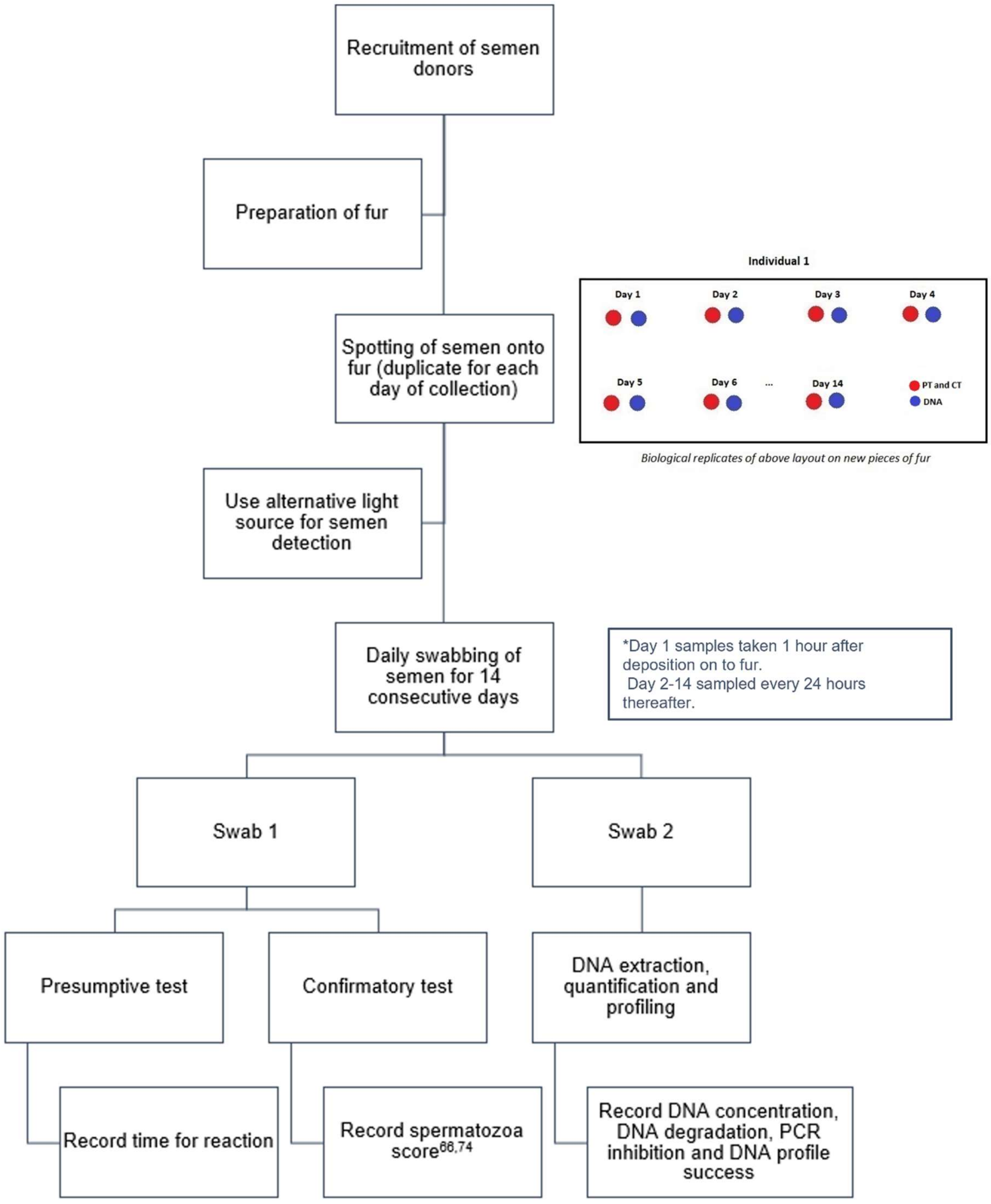

Figure S3: Flow diagram of experimental procedures.
